# Vision-related tasks in children with visual impairment: a multi-method study

**DOI:** 10.3389/fpsyg.2023.1180669

**Published:** 2023-07-13

**Authors:** Fatemeh Ghasemi Fard, Hooshang Mirzaie, Seyed Ali Hosseini, Abbas Riazi, Abbas Ebadi

**Affiliations:** ^1^Department of Occupational Therapy, University of Social Welfare and Rehabilitation Sciences, Tehran, Iran; ^2^Pediatric Neurorehabilitation Research Center, University of Social Welfare and Rehabilitation Sciences, Tehran, Iran; ^3^Department of Optometry, School of Rehabilitation Sciences, Iran university of Medical Sciences, Tehran, Iran; ^4^Behavioral Sciences Research Center, Life style institute, Nursing Faculty, Baqiyatallah University of Medical Sciences, Tehran, Iran

**Keywords:** children with visual impairment, vision-related tasks, OTPF-4, occupational therapy, multi-method study

## Abstract

**Objective:**

Functional Vision (FV) is vital for the successful growth of children with visual impairment. However, tasks related to measuring FV have not been thoroughly studied for this population. To address this gap, this study seeks to establish a comprehensive set of vision-related tasks that consider both the difficulty levels of activities and the ages of children with visual impairment.

**Methods:**

This study utilized a sequential multi-method design, including a scoping review, a qualitative content analysis, and a focus group. Firstly, a scoping review was conducted to identify vision-related tasks based on the literature. Then, to contextualize the vision-related tasks, a qualitative content analysis was carried out. Subsequently, a focus group was conducted to categorize the identified tasks based on their difficulty levels and the children’s level of dependency. We utilized the directed content analysis method to analyze the data, using the occupational domain of the Occupational Therapy Practice Framework 4th edition (OTPF-4) as the primary framework.

**Results:**

During the review phase, which included 22 studies, and the interview phase, which involved 16 participants, a total of 95 and 85 vision-related tasks were identified, respectively. These tasks were then categorized into 17 activities and five occupations, which included activities of daily living (ADL), instrumental activities of daily living (IADL), education, play, and participation in social activities. Among these occupations, ADL was the easiest, while participation in social activities was the most challenging. Finally, the tasks were arranged based on their difficulty level for children with visual impairment.

**Conclusion:**

A comprehensive list of vision-related tasks has been developed based on the difficulty level of the tasks and the degree of dependency of children with visual impairment. This list can be used to develop standardized instruments for assessing FV in children with visual impairment.

## Highlights

- The study identified vision-related tasks and categorized them under the components of the occupational domain of the Occupational Therapy Practice Framework 4th edition (OTPF-4).
- The identified tasks were then sorted based on their difficulty levels.
- The study categorized children with visual impairment into four age groups (0–3, 3.1–7, 7.1–10, and 10.1–16) based on their developmental stages to determine their level of dependency on vision-related tasks.

## 1. Introduction

Vision is a critical sensory function that is essential for a child’s overall neurodevelopment. It plays a crucial role in neuromotor, cognitive, and emotional development (Purpura and Tinelli, 2020). Any disruption in visual function, which pertains to the physiological aspects of the visual system, as well as functional vision (FV), which refers to the ability to use vision in practical tasks, has the potential to hinder the growth and development of children (Colenbrander, 2010; Silva et al., 2022). As visual function and FV require different assessment and management approaches (Purpura and Tinelli, 2020), this study specifically focused on the FV by exploring vision-related tasks.

Visual impairment is defined as a best-corrected visual acuity below 20/70 or a visual field of 10 degrees or less in the best eye (Prajna et al., 2015). The international classification of diseases 11th revision (ICD-11) (Pezzella, 2022) classifies the severity of visual impairment based on visual acuity as follows: no visual impairment (visual acuity ≤20/40), mild visual impairment (20/70 ≤ visual acuity < 20/40), moderate visual impairment (20/200 ≤ visual acuity < 20/70), severe visual impairment (20/400 ≤ visual acuity < 20/200), blindness (visual acuity of 20/1200 or counts fingers), blindness (light perception), and blindness (no light perception).

Globally, it is estimated that by 2050, 360 million and 474 million people will have mild and moderate-to-severe visual impairments, respectively (Bourne et al., 2020). In Iran, the prevalence of visual impairment was reported to be 5.57% in 2020 (Afarid et al., 2020).

Occupational therapy, occupation, activities, and tasks are all related concepts in occupational therapy, but they have different meanings and implications for the therapeutic process. Occupational therapy is a health profession that uses occupation as a therapeutic tool to promote health and well-being. Occupation encompasses all the activities people engage in to occupy themselves, including self-care, leisure pursuits, and meaningful contributions to their communities. Occupational therapists may use activities and tasks as therapeutic interventions to help clients develop skills, improve their physical and cognitive abilities, and promote overall well-being. Activities are purposeful actions related to a person’s interests and goals, such as cooking and exercising, while tasks are smaller units of action, such as pouring a glass of water or writing a letter (Thomas, 2012).

Occupational and rehabilitation professionals must identify limitations in FV and implement effective interventions to maximize it (Zemina et al., 2018). Although previous studies have emphasized examining FV in children with visual impairment to provide appropriate rehabilitation services (Colenbrander, 2010; Riazi et al., 2011; Silva et al., 2022), a comprehensive list of vision-related tasks for children with visual impairment has not been reported. Instruments have been developed to measure vision-related tasks in adults (Szlyk et al., 1990; Turco et al., 1994; Katsumi et al., 1995; Ross et al., 1999; van Dijk et al., 1999; Haymes et al., 2001), and in children with visual impairment (Gothwal et al., 2003; Khadka et al., 2010; Gothwal et al., 2012; Tadić et al., 2013; Pitakova and Zlatarova, 2018; Grumi et al., 2022; Wadhwani et al., 2022). However, the latter are self-reported and not age-specific. Our research indicates that Robertson et al. (2020) developed the only age-specific assessment tool for children between the ages of 8–18 with visual impairment. However, children under the age of 8 were excluded due to their inability to self-report (Robertson et al., 2020). Also, there are some lists of vision-related tasks available for adults, such as those in the International Classification of Functioning, Disability, and Health (ICF) (WHO, 2012) and Massof’s activity inventory (Massof et al., 2007). Massof et al. (2005b) developed a comprehensive functional assessment tool for adults, consisting of 337 vision-related tasks categorized into 41 activities and 3 occupations, namely: daily living, social interactions, and recreation. Each occupation represents a different set of activities, including daily living activities such as using public restrooms, maintaining personal hygiene, dressing, managing personal healthcare, eating, and other similar activities. Social interactions cover entertaining guests, preparing food for visitors, dining out, among others, and recreation encompasses leisure activities like sewing, needlework, knitting, crocheting, woodworking, metalwork, painting, drawing, and more. Subsequently, each activity represents a group of tasks (Massof, 2005b). In contrast, the ICF for Children and Youth (ICF-CY) is a classification tool specifically designed for children and youth aged from birth to 18 years. It provides a description of the functioning of children and youth from various perspectives, including body functions, anatomical characteristics, activities, and participation (WHO, 2007).

As previously indicated there is a dearth of comprehensive vision-related tasks and standardized tools available to assess FV in children with visual impairment. This can harm the successful management and rehabilitation of FV. Furthermore, the difficulty levels of vision-related tasks have not been determined based on children’s abilities, which can result in inaccurate outcomes in rehabilitation services (Gothwal et al., 2003; Colenbrander, 2010). Additionally, children’s age has not been considered, despite their varying levels of dependency on such tasks according to their developmental stage (Eccles, 1999; Keenan et al., 2016). Moreover, the utilization of the Occupational Therapy Practice Framework 4th edition (OTPF-4) can augment the credibility of the study (AOTA, 2020). Therefore, this study aimed to address the aforementioned gaps by exploring age-specified vision-related tasks in children with peripheral visual impairment based on task difficulty level, using the occupational domain of the OTPF-4. We specifically focused on the FV of children with peripheral visual impairment, as the type of injury and subsequent effects on FV may differ compared to cerebral visual impairment (Ortibus et al., 2019). To achieve the study’s occupations, a multi-method design was employed to gain a comprehensive and in-depth understanding of the phenomena under investigation (Chaboyer et al., 2004).

## 2. Materials and methods

### 2.1. Study design

**Figure 1:**
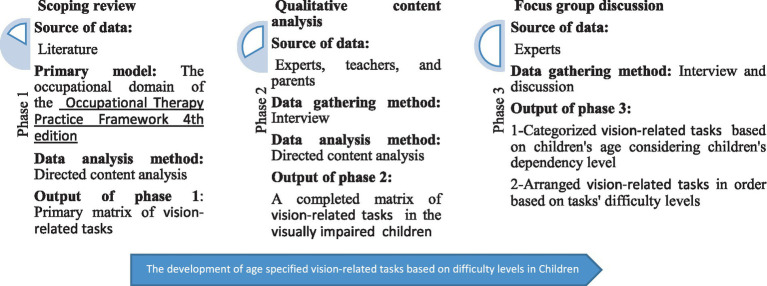
The three phases of the study.

The study utilized a sequential multi-method approach, which began with a scoping review, followed by a qualitative content analysis, and concluded with a focus group (Figure 1). Sequential multi-method is a research design that involves the use of multiple methods to investigate a single research question or hypothesis. This approach uses different research methods, such as literature review, interviews, and focus groups, in a specific sequence to complement each other’s results and build a more comprehensive understanding of the phenomenon under study (Subedi, 2016). This approach has been widely employed in prior studies to integrate scoping review data with qualitative data (Jolivet et al., 2021; Qiu et al., 2021; Claessens et al., 2022; Salifu et al., 2022).

Qualitative content analysis is a research method used to analyze large amounts of raw verbal data collected through interviews and condense them into categories or themes based on valid inference and interpretation via inductive or deductive reasoning (Mayring, 2004). The study, which is a subset of a larger study entitled “Developing an FV tool for children with visual impairment,” was conducted from September 2021 to September 2022 in Iran.

We used the occupational domain of OTPF-4 as the study framework which includes several occupations such as activities of daily living (ADL), instrumental activities of daily living (IADL), health management, rest and sleep, work, education, play, leisure, and social participation (AOTA, 2020). Moreover, in line with the works of Thomas (2012) and the American Occupational Therapy Association (AOTA) (Thomas, 2012; AOTA, 2020), the present study categorized vision-related tasks into three levels: tasks as the lowest level, activities as a group of tasks, and occupations as broad categories of activities such as daily living, social, recreational, and educational activities.

### 2.2. Scoping review

#### 2.2.1. Participants and sampling

In the review phase, all relevant articles published in international journals, as well as grey literature, were included. The inclusion of grey literature ensures that researchers do not overlook relevant information and can obtain a more complete picture of the current state of knowledge on a topic. Table 1 outlines the eligibility criteria for each part of the study participants.

**Table 1:** The inclusion and exclusion criteria for each part of the study participants.

Study’s phase	Inclusion criteria	Exclusion criteria
Scoping review	Articles (primary or secondary studies) with quantitative or qualitative design Published in a full text in English or Persian language in the peer review journals Examining FV and vision-related tasks in children with visual impairment with peripheral vision disorder under 16-year-old [not subjects with other impairments (such as hearing loss or intellectual impairment)] Limiting to visual impairments ranging from moderate to extremely severe according to ICD 10 Grey literature Published until September 2022	Written in languages other than English or Persian Unrelated/unavailable full-text studies Focusing on blindness and light perception, or cerebral vision disorder
Qualitative content analysis	Being an occupational therapist, teacher or parent of children with visual impairment with peripheral vision disorder (<16-year-old) and moderate to extremely severe visual impairment (according to ICD 10) Having experience in working with children with visual impairment with moderate to extremely severe visual impairment (according to ICD 10) for at least 1 year Having a willingness to participate in the study	Unwillingness to follow the study Working only with children who are blind or only light perception Working with children >16-year-old
Focus group	Having relevant experience or knowledge Having diversity in terms of work setting (academic, occupational, and rehabilitative settings) and years of experience Being available willingness to participate providing informed consent	Having a conflict of interest or any biases to the results of the study

#### 2.2.2. Data collection

In the review phase, we conducted thorough searches on electronic databases including PubMed, Scopus, Web of Science, and Cochrane, as well as on internal databases such as Magiran, SID, Irandoc, and Elmnet from the time of inception until April 2022. Our search strategy involved the use of both Medical Subject Heading (MeSH) and non-MeSH terms. Additionally, we reviewed relevant search results on Google and Google Scholar. To access research materials that are not traditionally published in academic journals or books, such as reports, theses, dissertations, conference proceedings, and government documents, we also searched grey literature. Furthermore, we screened the reference lists of the included articles to identify more studies and conducted hand-searching for a few key journals. The keywords and search strategies are provided in Supplementary file S1. Two investigators (F.G. and H.M.) screened the titles and abstracts of related studies, followed by reading the full texts. Any uncertainties were resolved through discussion. We utilized a data form to gather relevant information, including author name, year of study, country of origin, study design, age of participants (in years), visual status, and type of instrument used. Quality assessment was not performed, since it was not necessary for scoping review (Levac et al., 2010; Peters et al., 2015).

#### 2.2.3. Data analysis

The data were analyzed using Elo and Kyngäs’ method (Elo and Kyngas, 2008), by two authors (F.G. and H.M.), considering the occupational domain of the OTPF-4 as the primary framework. To report the results, we organized vision-related tasks identified from the literature into subdomains of the occupational domain of the OTPF-4 and constructed a primary data matrix.

#### 2.2.4. Trustworthiness

The scoping review was guided by the PRISMA extension for scoping reviews (PRISMA-ScR) (Tricco et al., 2018) (Supplementary file S3).

### 2.3. Qualitative content analysis

#### 2.3.1. Participants and sampling

In the qualitative content analysis phase, we conducted in-depth individual interviews with a purposive sample of participants that included teachers and parents of children with visual impairment, as well as rehabilitation experts. The participants were recruited from schools of children with visual impairment and rehabilitation centers in Tehran, Iran. To identify potential participants, we contacted these centers via email or phone, provided them with detailed information about the study, and asked for their assistance in identifying eligible participants. We used purposive sampling to ensure that the participants had diverse backgrounds and experiences and could provide rich and varied perspectives on the topic under investigation.

#### 2.3.2. Data collection

To collect rich and valid data, we utilized a combination of semi-structured, in-depth, face-to-face interviews, descriptive writing, and field notes. The interviews were conducted by two authors, F.G. (a doctoral student of occupational therapy) and H.M. (an associate professor of occupational therapy), both of whom possess over 10 years of research experience. The interviews were conducted between May and July 2022. The researchers explained the study’s purpose and obtained permission to record the interviews, as well as the possibility of a re-referral to confirm the findings. We used a semi-structured interview guide (Supplementary file S3), which was followed by more exploratory questions to obtain deep and rich data. Individual interviews lasted 40–60 min and were conducted in a quiet and private location, either in-person or remotely, depending on the participants’ preferences and availability. The interviews were audio-recorded with the participants’ consent. We continued conducting individual interviews until we reached data saturation, which occurs when information is repeated, and no further new information is obtained (Fusch and Ness, 2015). The researchers also took descriptive notes during the interviews and wrote field notes immediately after the interviews to capture their initial thoughts and impressions. The notes were used to supplement the data collected from the interviews and to aid in the analysis process.

#### 2.3.3. Data analysis

During the interview phase, audio recordings were transcribed verbatim and then carefully reviewed multiple times to identify meaning units and codes. The codes were then sorted and added to a primary matrix based on their similarities and differences. Any codes that did not align with the subdomains of the occupational domain of OTPF-4 were considered for new categories (Lewis, 2018). MAXQDA software version 10 was used for data analysis. The analysis process was iterative, with the researchers constantly refining and revising the codes and categories to ensure accuracy and rigor. The analysis was performed by two researchers independently and any discrepancies were discussed and resolved through consensus.

#### 2.3.4. Trustworthiness

The qualitative aspect of the study adhered to the Consolidated Criteria for Reporting Qualitative Research (COREQ) guidelines (Tong et al., 2007), which are outlined in Supplementary file S4. In addition, Lincoln and Guba’s four criteria, including credibility, dependability, confirmability, and transferability, were used to establish trustworthiness. To maintain credibility, we utilized triangulation of data sources, peer review, and engaged with the study for over 8 months. Co-author checks and member checks were also conducted to establish dependability. Furthermore, all study steps were thoroughly documented to ensure confirmability, allowing others to track all research-related activities. To ensure transferability, we clearly described the data collection, data analysis, and sample settings. This allowed readers to understand the context and setting of the study and assess whether the findings could be applied to other settings or populations.

### 2.4. Focus group discussion

#### 2.4.1. Participants and sampling

The focus group discussion was conducted with a select group of experts in occupational therapy, consisting of professors with specialized knowledge in the field, as well as a visual expert with relevant expertise.

#### 2.4.2. Data collection and analysis

The focus group consisted of two consecutive sessions, each lasting one and a half hours. In these sessions, a panel of experts evaluated the expected level of dependence of children with visual impairment in performing specific tasks across 17 activities, grouped into four age categories (0–3, 3.1–7, 7.1–10, and 10.1–16). The experts were also asked to rank the difficulty of these tasks, taking into account factors such as the severity of the visual impairment, the use of non-visual senses, environmental conditions, and the distance between the children and the objects of interest. Participants were allowed to consult with one another as needed, and all findings were carefully recorded.

#### 2.4.3. Data analysis

The focus group recordings were transcribed verbatim. The expected level of dependence of children with visual impairment was then assessed and ranked on a four-point scale, including high dependence, low dependence, independent, and high independence, for each of the previously mentioned age groups. In addition, the tasks were ranked in terms of difficulty.

## 3. Results

### 3.1. Characteristics of samples

#### 3.1.1. Phase 1: characteristics of primary studies

**Figure 2:**
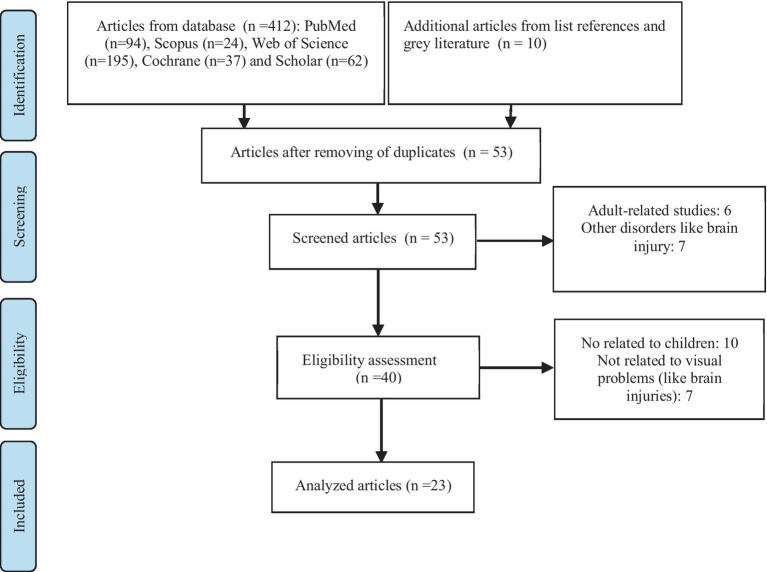
Study selection process.

In the scoping review, 422 articles were initially identified as potentially relevant. After removing duplicate and unavailable records, the titles and abstracts of 53 articles were reviewed. Subsequently, 40 studies underwent full-text assessment, and ultimately, 23 articles met the inclusion criteria. The study selection process is illustrated in Figure 2.

All studies were published between 2003 and 2022 and originated from various countries, including the United Kingdom (Khadka et al., 2010; Tadić et al., 2013, 2017; Dahlmann-Noor et al., 2017; Tailor et al., 2017; Dahlmann-Noor et al., 2018; Elsman et al., 2019b; Robertson et al., 2020, 2021, 2022), the United States (Castañeda et al., 2016; Kaiser and Herzberg, 2017; Hatt et al., 2019, 2020; Leske et al., 2019, 2021), India (Gothwal et al., 2003, 2012; Nirmalan et al., 2004), Turkey (Çakmak et al., 2016; Tunay et al., 2016), and Bulgaria (Pitakova and Zlatarova, 2018). Supplementary file S5 provides detailed characteristics of the studies.

#### 3.1.2. Characteristics of participants in the section of qualitative content analysis

Sixteen participants took part in the interview phase, comprising nine teachers from low-vision schools, four occupational therapists, and three parents. On average, the interviews lasted approximately 45 min. The majority of participants (75%) were female and the average age range was 40–45 years old. Table 2 presents detailed information about the participants.

**Table 2:** Participants’ characteristics.

Participants’ code	Age	Gender	Type of profession	Time of experience (years)	Number of interviews	Interview length (minutes)
1	30	Female	Teacher	7	1	45
2	40	Male	Teacher	8	2	44
3	52	Female	Teacher	10	1	48
4	60	Male	Teacher	18	1	36
5	35	Female	Teacher	15	1	40
6	33	Male	Teacher	20	2	42
7	41	Female	Teacher	25	1	45
8	46	Female	Teacher	23	1	42
9	39	Male	Teacher	19	1	45
10	38	Female	Occupational therapist	15	1	44
11	50	Male	Occupational therapist	28	1	49
12	48	Female	Occupational therapist	25	1	47
13	32	Male	Occupational therapist	9	2	44
14	36	Male	Father	20	1	43
15	45	Female	Mother	18	1	46
16	41	Female	Mother	12	2	45

#### 3.1.3. Characteristics of experts in the focus group discussion

The focus group consisted of five occupational and visual therapists, all of whom held qualifications as either full or associate professors. Out of the five expert participants, two were female and the remaining three were male.

### 3.2. Vision-related tasks yielded from scoping review and interviews

The scoping review and qualitative content analysis initially identified 496 vision-related tasks. After integrating similar ones, a total of 180 tasks remained, categorized under the following five occupations: ADL, IADL, education, play, and participation in social activities (Table 3). No codes emerged for other components of the occupational domain of the OPTF-4, including health management, rest and sleep, leisure, and work. A narrative description of each occupation and activity is outlined as follows:

**Table 3:** Dependency level of children with visual impairment based on their age groups in each row of occupations, activities, and tasks.

Occupations	Activities	Number of tasks		The dependency levels based on the age groups (years)			
		Scoping review	Qualitative content analysis	0–3	3.1–7	7.1–10	10.1–16
Activities of daily living	Dressing	1	7	HD^†^	LD^†^	I^†^	HI^†^
	Feeding	–**	5	HD	HD	LD	HI
	Personal hygiene and grooming	5	8	HD	HD	HD	I
	Toilet hygiene	–	10	HD	HD	I	HI
Instrumental activities of daily living	Meal preparation	3	10	HD	HD	LD	I
	Using electronic devices	2	5	HD	HD	HD	I
	Shopping	10	7	HD	HD	HD	LD
Education	Face/object recognition	5	1	HD	LD	HI	HI
	Participation in class	5	–	HD	HD	I	HI
	Mobility	2	1	HD	HD	I	I
	Writing and drawing	4	9	HD	HD	I	I
	Reading	5	1	–	HD	LD	I
Play	Indoor activities	12	12	HD	LD	I	HI
	Outdoor activities	5	4	HD	HD	I	I
Participation in social activities	Virtual activities	18	1	HD	HD	LD	I
	In-person activities	–	4	HD	HD	LD	I
	Community mobility	18	–	HD	HD	HD	LD

^†^HD, high dependence; LD, low dependence; I, independent; HI, high independence; **, not achieved.

#### 3.2.1. ADL

ADL refer to self-care tasks that children with visual impairment should perform to take care of themselves. These activities were classified into four activities: dressing, feeding, personal hygiene and grooming, and toilet hygiene. The description of each goal is as follows:

##### 3.2.1.1. Dressing

Dressing and undressing multiple times per day is an important ADL for children with visual impairment. Studies have reported that lacing shoes is one dressing task (Gothwal et al., 2003; Nirmalan et al., 2004; Gothwal et al., 2012; Ganesh et al., 2013; Pitakova and Zlatarova, 2018).

According to participants’ data, children with visual impairment often need to locate clothes and accessories in closets and drawers, put them on or remove them, and fasten and adjust their clothing and shoes. However, dressing independently can be extremely challenging for children with severe visual impairments. Some participants explained:

*“Some routine dressing activities are wearing clothes or taking them off; tying a scarf…” – P 1, teacher.*

*“Finding clothes that are suitable and accessible can be very challenging for near-to-blind cases. Many others struggle with buttons, zippers, and snaps.” – P 12, occupational therapist.*

*“Putting on clothes the right way, fixing mistakes, and making adjustments independently is challenging for most children with visual impairment.” – P 16, parent.*

##### 3.2.1.2. Feeding

A qualitative study revealed that a 15-month-old infant with visual impairment independently fed herself small pieces of avocado while seated in a high chair. She picked up her fork and used her hand to place a tiny piece of food on the tines, and then brought the fork to her mouth to take a bit (Smyth et al., 2014).

According to participants’ experiences, children with visual impairment can name food on a plate, pour water into a glass without spilling, and eat without dropping food, although the quality of their task may vary. Regarding feeding a participant described how children with visual impairment eat independently:

*“To feed themselves, mild to moderate children with visual impairment typically sit at the dining table and use a spoon and fork to eat food or fill a glass to drink water.” – P 3, teacher.*

Moreover, a parent explained:

*“My son is becoming increasingly independent with feeding himself. He can recognize the most common foods by touch and smell. However, identifying utensils and holding them properly is still challenging for him.” – P 16, parent.*

##### 3.2.1.3. Personal hygiene and grooming

Several studies reported vision-related tasks related to personal hygiene and grooming, including: combing or trimming hair; shaving and removing body hair using a laser or tweezers (especially for older children ages 15–18); applying and removing cosmetics by girls (Tadić et al., 2013, 2017; Elsman et al., 2019b; Robertson et al., 2020); applying paste to a toothbrush and brushing teeth; and identifying dirty stains on clothes (Gothwal et al., 2003, 2012; Nirmalan et al., 2004; Ganesh et al., 2013; Pitakova and Zlatarova, 2018).

A few participants additionally mentioned tasks like cleaning glasses, arranging scarves, flossing teeth, and trimming nails. For example:

A teacher explained:

*“some severely visually impaired students find basic tasks like brushing their hair thoroughly to be challenging, while others can do them easily.”* - P8, teacher.

A mother described:

*“My son cleans his glasses as necessary.”* –P 16, parent.

Another teacher said:

*“Here, we see that low vision girls check their scarves to make sure they are arranged properly.”* –P 7, teacher.

##### 3.2.1.4. Toilet hygiene

Only interview data revealed various vision-related tasks regarding toileting, such as: finding the bathroom, locating the toilet seat, preparing, sitting, and using the toilet, rinsing the toilet or water closet, flushing, finding and using the sink, locating soap, washing hands, finding toilet paper or paper towels, and drying hands with a towel or tissue. For instance, two participants said:

“*My daughter needs assistance finding the toilet and sink in unfamiliar bathrooms. At home, she knows her way around but still struggles with tasks like aiming into the toilet and washing her hands properly.*” – p 16, parent.

“*Toileting consists of multiple sub-tasks that can be performed independently or dependently by these kids”* – P. 14, occupational therapists.

#### 3.2.2. IADL

Participants and literature indicate that children with visual impairment perform more complex activities beyond ADL. This domain consists of three activities: “meal preparation,” “using electronic devices,” and “shopping,” which are described below:

##### 3.2.2.1. Meal preparation

Depending on their capabilities, children with visual impairment may prepare and serve meals, identify foods’ nutritional value, clean food, and wash dishes (Tadić et al., 2013, 2017; Elsman et al., 2019b; Robertson et al., 2020).

According to participants’ experiences, children with visual impairment can peel fruits and vegetables like potatoes. They are also able to locate food items and utensils, pour or mix food materials without spilling, read package labels, and judge the preparedness of foods. According to the participants, children’s ability to complete these tasks depends on their age, with older children typically performing them more easily than younger ones. Two occupational therapists stated:

*“My 7-year-old case struggles with meal preparation tasks, while my other 10-year-old case can make sandwiches, slice apples, and open most food packages independently.”* – P 13, occupational therapist.

*“Even simple meal preparation tasks like peeling a cucumber, cutting an orange in half, or slicing a potato into sticks are within the capabilities of many children with visual impairment. With the proper adaptive tools and techniques, and some guidance initially, they can accomplish independent meal preparation activities that are appropriate for their age and development.”*– P 5, occupational therapist.

##### 3.2.2.2. Using electronic devices

Children with visual impairment can participate in activities involving technology, such as turning a television on and off or changing channels (Gothwal et al., 2003, 2012; Nirmalan et al., 2004; Ganesh et al., 2013; Dahlmann-Noor et al., 2017, 2018; Pitakova and Zlatarova, 2018). They can also use home computers for online learning (Tadić et al., 2013, 2017; Elsman et al., 2019b; Robertson et al., 2020).

Moreover, many participants mentioned that children with visual impairment use smartphones or tablets, especially during COVID-19 when virtual learning occurred via these devices. Other tasks were also reported, such as dialing phone numbers, researching phone numbers, watching television, listening to music while wearing headphones, and understanding stories in movies. However, additional support and accommodations from teachers and parents may still be necessary, depending on the severity of a child’s visual impairment. For instance, two participants stated:

*“Most visually impaired students like to use their iPad to listen to music and play games. As they have gotten older, they learned how to navigate the device more independently.”* – P 9, teacher.

*“With tools like screen readers, text enlargement software, and audio descriptions, visually impaired students can navigate online learning interfaces, read assigned readings, watch instructional videos, and complete and submit assignments independently.”* – P 15, teacher.

##### 3.2.2.3. Shopping

According to the literature, children with visual impairment can go grocery shopping. At stores, they can read signs and posters; at restaurants, read menus and price tags, at pharmacies, select a payment method and complete transactions (Gothwal et al., 2003, 2012; Nirmalan et al., 2004; Ganesh et al., 2013; Tadić et al., 2013, 2017; Çakmak et al., 2016; Dahlmann-Noor et al., 2017, 2018; Pitakova and Zlatarova, 2018; Elsman et al., 2019b; Robertson et al., 2020).

Furthermore, participants mentioned additional tasks like locating products in the store, navigating an outdoor store independently, and walking around a store. One mother said:

*“My 14-year-old son needs assistance finding specific items in big stores, but in our neighbourhood market she knows where everything is and can get what we need on her own.”* – P 16, mother.

#### 3.2.3. Education

The education occupation was made of various activities including reading, face and object recognition, participating in class, mobility, writing and drawing, and reading. These activities are explored in detail below:

##### 3.2.3.1. Face/object recognition

Several studies found that children with visual impairment can distinguish their peers and teachers by face. Despite their visual impairment, these children can also find their belongings within the classroom. However, their ability to recognize faces and find objects greatly depends on their visual acuity (Gothwal et al., 2003, 2012; Nirmalan et al., 2004; Ganesh et al., 2013; Tadić et al., 2013, 2017; Çakmak et al., 2016; Dahlmann-Noor et al., 2017, 2018; Pitakova and Zlatarova, 2018; Elsman et al., 2019b; Robertson et al., 2020).

Moreover, teachers agreed that visually impaired students exhibited varying levels of accuracy in identifying objects in the classroom, such as balls or colors. For instance, a participant said:

*“‘Students with milder low vision can identify simple shapes and objects, while those with more severe vision loss often require physical or verbal prompts.”* – P 7, teacher.

##### 3.2.3.2. Participation in classes

According to a literature review, children with visual impairment attend a variety of classes including science, geography, math, physical activity, English/Dutch, and gymnastics (Gothwal et al., 2003, 2012; Nirmalan et al., 2004; Colenbrander, 2010; Ganesh et al., 2013; Tadić et al., 2017; Tailor et al., 2017; Pitakova and Zlatarova, 2018; Hatt et al., 2019).

The interview data did not suggest any new tasks that children with visual impairment perform in the classroom setting.

##### 3.2.3.3. Mobility

Several studies found that children with visual impairment can walk around their school independently or with assistance (Tadić et al., 2013, 2017; Elsman et al., 2019b; Robertson et al., 2020) as well as navigate hallways without colliding with people or objects (Gothwal et al., 2003; Nirmalan et al., 2004; Ganesh et al., 2013). Besides, from the perspective of a participant, children with visual impairment can walk outside of school with or without assistance. A teacher said:

*“Under the supervision and guidance of teachers, our visually impaired students navigate the building while learning how to safely avoid obstacles.”* – P 3, teacher.

##### 3.2.3.4. Writing, drawing, and coloring

Current evidence has demonstrated that children with visual impairment are capable of writing on blackboards, taking notes from their peers’ notebooks, drawing, coloring, and sorting information (Gothwal et al., 2003, 2012; Nirmalan et al., 2004; Colenbrander, 2010; Ganesh et al., 2013; Tadić et al., 2017; Tailor et al., 2017; Pitakova and Zlatarova, 2018; Hatt et al., 2019). They also use computers when doing assignments at school (Tadić et al., 2013, 2017; Elsman et al., 2019b; Robertson et al., 2020).

Furthermore, according to some parents and teachers, some children with visual impairment can type their homework themselves using laptops or computers. One teacher said:

*“My visually impaired students need enlarged text and speech output on their laptops, tablets, and computers. Once they have the right adaptive technology, they can complete written assignments just like their sighted peers.”* –P 7, teacher.

##### 3.2.3.5. Reading

Reading tasks for children with visual impairment included reading textbooks, worksheets, exams, handwriting, and information on the board in the classroom (Gothwal et al., 2003, 2012; Nirmalan et al., 2004; Ganesh et al., 2013; Tadić et al., 2013, 2017; Çakmak et al., 2016; Dahlmann-Noor et al., 2017, 2018; Pitakova and Zlatarova, 2018; Elsman et al., 2019b; Robertson et al., 2020).

However, participants reported that many children with visual impairment struggled with reading tasks and offered strategies to make them more achievable, such as using enlarged print, high-quality photocopied materials, braille font, magnified electronic text with reduced glare, or audio books. One participant stated:

*“Reading from regular print sources can be frustrating for extreme low-vision cases; while Adjusting text size, font, and color contrast can make a huge difference for my visually impaired students when they are doing independent reading.”* – P 9, teacher.

#### 3.2.4. Play

Play was another occupation, with two activities: indoor and outdoor play activities.

##### 3.2.4.1. Indoor activities

Children engage in indoor play activities at home for entertainment. According to the literature, some of the play activities that children with visual impairment participate in include watching television, playing video and computer games, and drawing or painting (Tadić et al., 2013, 2017; Elsman et al., 2019b; Robertson et al., 2020), and listening to music (Gothwal et al., 2003, 2012; Nirmalan et al., 2004; Ganesh et al., 2013; Tadić et al., 2013, 2017; Çakmak et al., 2016; Dahlmann-Noor et al., 2017, 2018; Pitakova and Zlatarova, 2018; Elsman et al., 2019b; Robertson et al., 2020).

Further, interviews identified additional activities like solving puzzles, playing with talking dolls, and braille sudoku puzzle games. For example, a participant stated:

*“My son loves playing puzzle games, ball games, and hopscotch and listening to audio storybooks at home.” – p 14, A father.*

##### 3.2.4.2. Outdoor activities

Studies have identified various items that include mutual play, team games such as football, hide-and-seek, and playing tennis or cricket (Tadić et al., 2013, 2017; Elsman et al., 2019b; Robertson et al., 2020).

One participant reported that outdoor activities included games such as tennis, hopscotch, playing with balls or marbles, and shadow games. Two other participants also mentioned.

*“Some children with visual impairment in my program enjoy outdoor games like hopscotch, playing with balls, marbles, or shadows.” – P 11, occupational therapist.*

*“Here in our school, some low-vision kids even enjoy playing tennis” – P 7, teacher.*

#### 3.2.5. Participation in social activities

Social participation involves interactions with family, friends, peers, and community members. This occupation was made up of three activities: virtual activities, in-person activities, and community mobility.

##### 3.2.5.1. Virtual activities

Studies found that children with visual impairment primarily participate in virtual social activities through smartphones and apps like Facebook and Twitter (Gothwal et al., 2003, 2012; Nirmalan et al., 2004; Ganesh et al., 2013; Tadić et al., 2013, 2017; Çakmak et al., 2016; Dahlmann-Noor et al., 2017, 2018; Pitakova and Zlatarova, 2018; Elsman et al., 2019b; Robertson et al., 2020).

Interview results confirmed that children with visual impairment communicate primarily in virtual forms. A parent stated:

*“My son uses social media applications and text messaging on his smartphone to stay in contact with his friends regularly. Since he has low vision, the enlarged text options, light or dark color themes, and voiceover accessibility features on his apps allow him to communicate independently through social media “*– P 16, parent.

##### 3.2.5.2. In-person activities

According to primary research, children with visual impairment can participate in academic classes and engage in general conversations with classmates, teachers, and peers both inside and outside of the classroom. However, the difficulty in recognizing facial expressions or identifying individuals, particularly among those with severe visual impairment, can have a significant impact on their social interactions and relationships (Tadić et al., 2013, 2017; Elsman et al., 2019b; Robertson et al., 2020).

Interviews with participants did not reveal additional codes for this domain.

##### 3.2.5.3. Community mobility

Children with visual impairment frequently utilize public transportation, such as trains and busses, to visit places like the cinema or theater, often with the assistance of parents or caregivers. They may also navigate through unfamiliar areas, using elevators, crossing the street, and walking on uneven roads with assistance. Furthermore, when accompanied by family members, children with visual impairment can navigate around obstacles and avoid other people, ascend and descend stairs, and detect moving objects like cars (Gothwal et al., 2003, 2012; Nirmalan et al., 2004; Ganesh et al., 2013; Tadić et al., 2013, 2017; Çakmak et al., 2016; Dahlmann-Noor et al., 2017, 2018; Pitakova and Zlatarova, 2018; Elsman et al., 2019b; Robertson et al., 2020).

No additional tasks were identified during the interview phase.

### 3.3. Classifying vision-related tasks based on children’s age

Experts suggest that children with visual impairment may perform vision-related tasks at varying levels of dependency depending on their age. In the present study, the children’s ages were classified into four developmental stages: 0–3, 3.1–7, 7.1–10, and 10.1–16, based on previous research (Eccles, 1999; Keenan et al., 2016) and the opinions of the focus group. The ability of children to perform vision-related tasks was categorized as high dependence (HD), low dependence (LD), independence (I), or high independence (HI), according to these age groups.

### 3.4. Vision-related tasks based on the difficulty levels

The vision-related tasks identified across the 17 activities were ranked according to their level of difficulty. Factors such as the child’s environment, the distance from an object, the involvement of non-visual senses, and the degree of visual impairment were taken into account during the sorting process. Experts suggested that children with visual impairment may find indoor vision-related tasks easier than outdoor ones, as they are more familiar with indoor environments. As a result, ADL tasks were considered the easiest, while social participation tasks were viewed as the most challenging, as shown in Table 4.

**Table 4:** Arrangement of vision-related tasks based on their difficulty levels in 17 activities.

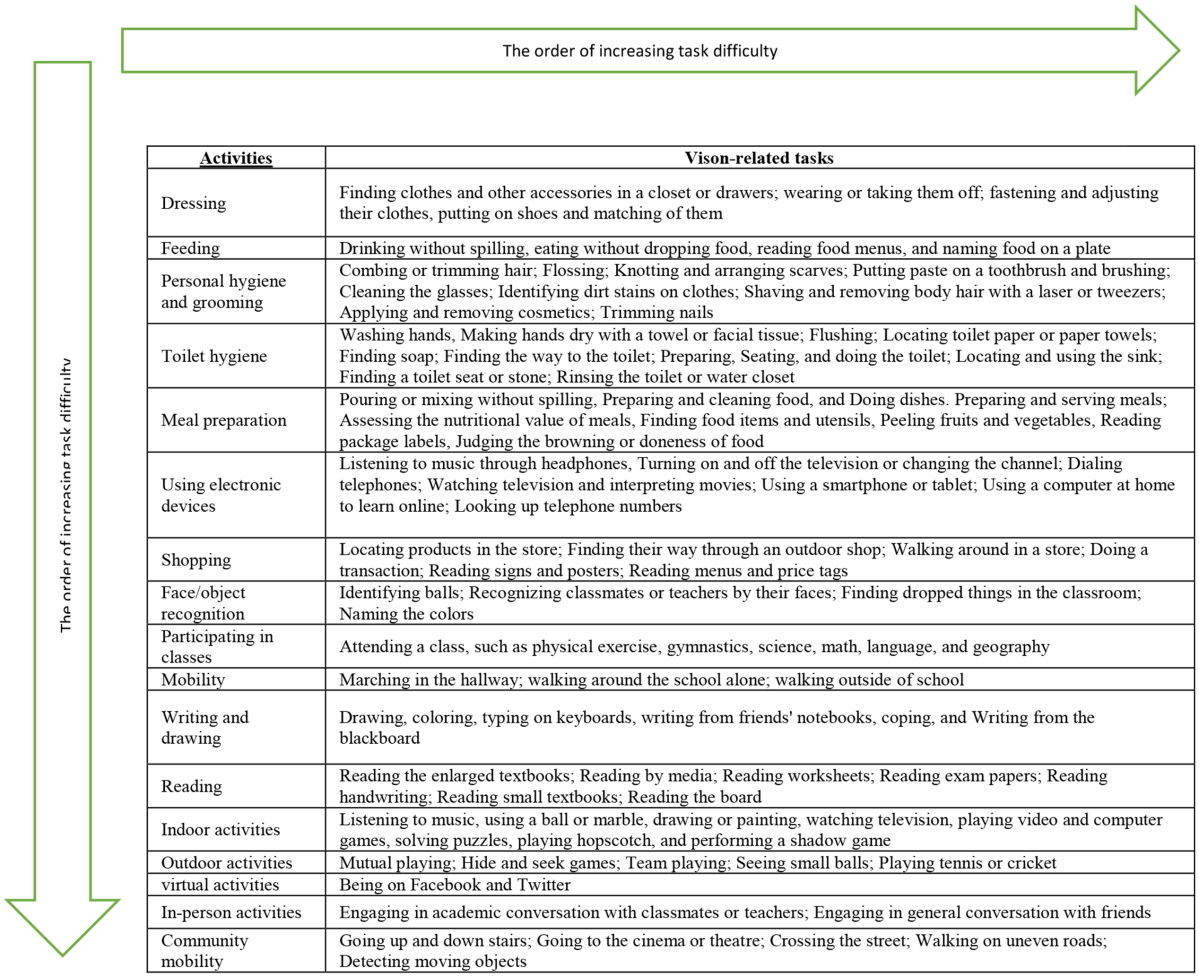

## 4. Discussion

Visual impairment has been shown to have a negative impact on children’s learning and daily activities, according to multiple studies. For instance, one study revealed that teachers can gain insights into students’ reading difficulties, lack of motivation, low self-esteem, and difficulty concentrating by understanding the visual attributes and their impact on learning (Wilhelmsen and Felder, 2022). Other studies have indicated that children with visual impairment may face challenges in developing spatial abilities, including auditory and proprioceptive spatial localization, haptic orientation discrimination, and reach on sound (Cappagli et al., 2017, 2019). They may also experience delays in various motor abilities, as well as reduced walking speed due to a longer stance phase (Hallemans et al., 2011). Visual deprivation from birth to adulthood can cause functional abnormalities in the cortical areas responsible for cross-modal information processing. Specifically, visual impairment can affect multisensory processes and impede the exchange of sensory data during the exploration and identification of the immediate environment (Purpura et al., 2021).

However, an appropriate rehabilitation program can assist children with visual impairment to improve their growth and quality of life. For instance, a systematic review study revealed that sports camps, low vision device prescription and training can be effective in improving the functioning, participation, and quality of life of children with visual impairment (Elsman et al., 2019a). Another study found that perceptual development in individuals who are blind can be enhanced by utilizing auditory feedback that is naturally associated with bodily movements (Hallemans et al., 2011).

Given the importance of proper vision-related tasks for overall development (Prechtl et al., 2001; Ganesh et al., 2013), this study was conducted in three phases: a literature review, individual interviews, and a focus group. The occupational domain of the OPTF-4 was used as a framework. Vision-related tasks previously identified for children with visual impairment were extracted from the literature and organized into a primary data matrix based on subdomains. Context-specific tasks were then identified through qualitative content analysis and added to the matrix. Finally, the levels of dependence for different age groups of children with visual impairment in performing vision-related tasks were determined.

Our study identified five occupations, 17 activities, and 180 tasks. The occupations were comparable to those reported for adults by Massof et al. (2005a), except for vocational activities, which are typically performed by adults (Massof et al., 2005a). However, notable differences were observed in the level of activities and tasks. For instance, activities such as attending social functions, managing personal finances, dining out, and woodworking are beyond children’s abilities (Massof et al., 2007). Similarly, tasks such as hammering nails, writing checks, or taking pills are not expected of children. Examples of similar tasks between children and adults include finding clothes in the closet, finding soap or towels, or brushing their teeth (Massof et al., 2005b).

Based on our findings, children with visual impairment primarily performed ADL related to dressing, feeding, personal hygiene, grooming, and toilet hygiene. The ADL-related activities in our study were comparable to the subdomains in the OTPF, except for bathing, eating, functional mobility, and sexual activity (AOTA, 2020). Evaluation of ADL-related activities in children with visual impairments and implementation of habilitation practices, if necessary, is crucial. The Melbourne Low-Vision ADL Index is a test that can be used to evaluate ADL-related tasks in the general low-vision population (Haymes et al., 2001). Children with visual impairments may experience delays in acquiring the crucial skill of dressing due to the absence or reduction of visual input, as revealed in a study (Hayton et al., 2019). Therefore, training in independent living skills is a component of habilitation practice that aims to promote autonomy in children and adolescents with visual impairments, preparing them for adulthood (Hayton et al., 2019).

Our study identified three IADL-related activities for children with visual impairments: meal preparation, electronic device use, and shopping. The occupational domain of the OPTF-4 includes several subcategories related to caregiving, child rearing, driving, managing finances, maintaining safety, and expressing religious beliefs, which are typically associated with adults rather than children (AOTA, 2020). It’s important to acknowledge that children with visual impairments may have different abilities in these areas compared to typically developing children. They may require additional support and training to perform IADL independently.

This study found that children with visual impairment primarily perform tasks related to education, such as face and object recognition, class participation, mobility, writing, drawing, and reading. However, they may encounter difficulties accessing and leaving school, reading, writing, and navigating obstacles such as ramps and slopes on sidewalks (Taşkin et al., 2020). Students with visual impairment may access information through Braille, audio-tape, or enlarged print, and may require additional time to process information (Lombardi, 2021). Effective management of visual limitations can improve educational success, and interventions such as assistive technology, modified learning environments, and specialized instruction or support can be beneficial (Maćkowski et al., 2022). Teachers’ attitudes toward students with disabilities in inclusive schools can influence their academic success, and it is crucial to ensure that teachers are supportive and accommodating (Temesgen, 2018). However, a survey conducted in Ethiopia revealed that teachers were reluctant to care for disabled children (Dagnew, 2013). Vision screening by teachers in schools is also important to ensure that children with visual impairments receive the necessary support to achieve their academic potential (Wilhelmsen and Felder, 2022).

Additionally, our study found that children with visual impairment enjoy playing both indoors and outdoors. Play is crucial for developing social-emotional, cognitive, and physical skills, and parents and peers can play a significant role in promoting appropriate play (Sacks et al., 1992). Children with visual impairment spend a higher proportion of their playtime alone compared to their typically developing peers (Schneekloth, 1989). They also tend to interact more with adults and have limited experiences with complex or social games that involve rules or creativity (Rettig, 1994; Tzvetkova-Arsova and Zappaterra, 2017). Therefore, it is important to support and encourage developmentally appropriate play for children with visual impairment.

Participation in social activities among children with visual impairment involved virtual and in-person activities, as well as community mobility. Our findings showed that community participation for these children mostly relied on mobile devices and social media. Digital technologies, along with entertainment features, can help meet some of their social needs, such as connecting with friends and classmates virtually (Pacheco et al., 2018). Children with visual impairment tend to participate virtually in social activities and have friends from school or relatives, as interacting with people outside their virtual world may pose systemic barriers such as discrimination or bullying (Woodgate et al., 2020). A recent study developed the Visual Impairment Developmental Autonomy (VIDA) scale to evaluate children’s autonomy in areas such as mobility, communication, daily living skills, socialization, and learning, using patient and parent-reported outcome measures (Grumi et al., 2022). However, unlike our study, this study focused on children with peripheral visual impairment who were blind or severely visually impaired.

Our study found that children with visual impairment perform indoor activities, such as ADL, more easily than outdoor activities, likely due to the controlled and familiar indoor environment. Similarly, other studies have shown that the environment plays a significant role in the vision-related tasks of children with visual impairment, particularly when tasks involve senses other than vision, such as touch (Leffert and Jackson, 1998; Gur, 2014).

Our study developed a dependence continuum for vision-related tasks based on the age spectrum of children with visual impairment. While previous studies did not determine how to divide these activities, Alexandrea et al. measured vision-related tasks overall for children aged 8–18 (Robertson et al., 2020). In our study, experts in focus groups agreed that there is no rigid boundary based on children’s ages, and the division is based on developmental stage and the complexity of vision-related tasks. Younger children may require more assistance to complete basic vision-related tasks independently, while older children may perform more complex tasks with reduced dependence. However, the relationship between age, developmental stage, and independence in performing vision-related tasks is complex and individualized for each child based on their visual impairment, comorbidities, and environment. Future research exploring this dependence continuum for children with visual impairment across developmental periods could provide valuable insights to optimize interventions that promote independence and quality of life.

This study presents, for the first time, a ranking system for vision-related tasks based on the task’s difficulty and the age group of children with visual impairment. Children with visual impairment experience more difficulty performing tasks that rely on vision compared to other senses, and they must learn to compensate for their visual deficits by using other skills such as hearing and touch. Reading is an example of a task that relies solely on vision and is particularly challenging for children with visual impairment (Gothwal et al., 2003). Factors such as the severity of the visual impairment, the utilization of non-visual senses, environmental factors, distance to objects, and self-esteem can affect children’s ability to perform vision-related tasks. Effective management of these factors can optimize the visual ability of children with visual impairment. Early intervention and personalized support that address each child’s unique abilities and needs may enhance their proficiency in performing vision-related tasks and lead to improved outcomes. Further research is necessary to validate this ranking system and identify the most effective targets for intervention to maximize independence in children with visual impairment.

### 4.1. Limitation

Our study exclusively focused on vision-related tasks in children with visual impairment, excluding those with total blindness or light perception vision. Additionally, this study only investigated vision-related tasks in children and no other age groups. Our sample may not have been representative of the population studied, and caution should be implemented when applying our findings to other contexts with different social, economic, and geographic characteristics due to the context-based nature of our interview data. However, we provided a comprehensive list of vision-related tasks for children with visual impairment under the age of 16 based on difficulty levels and the degree of independence for children with visual impairment, using a multi-method design and applying the OPTF-4 rehabilitation model.

## 5. Conclusion

Our study aimed to contribute to rehabilitation knowledge by identifying vision-related tasks according to difficulty levels and age groups of children with visual impairment, based on literature and participants’ viewpoints. We identified five occupations: ADL, IADL, education, play, and social participation, with education requiring the most vision-related tasks. Our study arranged the tasks by difficulty and dependency levels according to age groups, which can guide future studies in selecting task samples based on type, difficulty, and age. Our findings can inform rehabilitation programs and interventions for children with visual impairment, providing insight into specific vision-related tasks, their difficulty levels, and dependency levels. Healthcare professionals can tailor interventions to support FV, and our findings can be useful in developing assessment tools to evaluate FV and its progress over time in children with visual impairment. Currently, we are developing performance-based instruments to measure FV in this population.

## Data availability statement

The original contributions presented in the study are included in the article/Supplementary material, further inquiries can be directed to the corresponding author.

## Ethics statement

This study was approved by the Ethics Committee of the University of Social and Welfare Rehabilitation (USWR) with the ID of IR.USWR.REC.1400.187, Tehran, Iran. Written and verbal consent was obtained from all participants to record their interviews. Participants in the study were assured that their information would be kept confidential and they could withdraw from the study at any time.

## Author contributions

HM, FGF, SAH, AR, and AE: designing and performing tests, collecting the data, and co-writing the paper. HM and FGF: performing the analysis. HM, SAH, AR, and AE: supervising the research. All authors contributed to the article and approved the submitted version.

## Conflict of interest

The authors declare that the research was conducted in the absence of any commercial or financial relationships that could be construed as a potential conflict of interest.

## Publisher’s note

All claims expressed in this article are solely those of the authors and do not necessarily represent those of their affiliated organizations, or those of the publisher, the editors and the reviewers. Any product that may be evaluated in this article, or claim that may be made by its manufacturer, is not guaranteed or endorsed by the publisher.
