# Corrigendum: Vision-related tasks in children with visual impairment: a multi-method study

**DOI:** 10.3389/fpsyg.2023.1331551

**Published:** 2023-12-06

**Authors:** Fatemeh Ghasemi Fard, Hooshang Mirzaie, Seyed Ali Hosseini, Abbas Riazi, Abbas Ebadi

**Affiliations:** ^1^Department of Occupational Therapy, University of Social Welfare and Rehabilitation Sciences, Tehran, Iran; ^2^Pediatric Neurorehabilitation Research Center, University of Social Welfare and Rehabilitation Sciences, Tehran, Iran; ^3^Department of Optometry, School of Rehabilitation Sciences, Iran University of Medical Sciences, Tehran, Iran; ^4^Behavioral Sciences Research Center, Life Style Institute, Nursing Faculty, Baqiyatallah University of Medical Sciences, Tehran, Iran

**Keywords:** children with visual impairment, vision-related tasks, OTPF-4, occupational therapy, multi-method study

In the published article, there was an error in the Ethics statement as published. The ethics approval number was displayed as “IR.BMSU.REC.1399.518”, but should be “IR.USWR.REC.1400.187”. The corrected ethics statement appears below:

This study was approved by the Ethics Committee of the University of Social and Welfare Rehabilitation (USWR) with the ID of IR.USWR.REC.1400.187, Tehran, Iran. Written and verbal consent was obtained from all participants to record their interviews. Participants in the study were assured that their information would be kept confidential and they could withdraw from the study at any time.

Additionally, there was an error in the affiliations as published. The affiliation for author Hooshang Mirzaie was incorrectly noted as affiliation 1 and their correct affiliation was missing from the affiliation list. This affiliation should be listed as affiliation 2, “Pediatric Neurorehabilitation Research Center, University of Social Welfare and Rehabilitation Sciences, Tehran, Iran”, with the numbering of the remaining affiliations adjusted accordingly.

The authors apologize for this error and state that this does not change the scientific conclusions of the article in any way. The original article has been updated.

