# Supplementary material for: Vision-related tasks in children with visual impairment: a multi-method study
Source: Front Psychol. 2023 Jul 13;14:1180669. doi: 10.3389/fpsyg.2023.1180669 (PMC10374319; doi:10.3389/fpsyg.2023.1180669)
Supplement: Supplementary file 1 [file Data_Sheet_1.docx]

Supplementary File 1: Search strategy and the number of papers achieved from each database

| **Database** | | **Search Strategy** | **Number** |
| --- | --- | --- | --- |
| PubMed | | "child"[MeSH Terms] AND ("visual impairment"[All Fields] OR "low vision"[All Fields] OR ("visually impaired persons"[MeSH Terms] OR "blindness"[MeSH Terms])) AND ("functional vision"[All Fields]) | 94 |
| Scopus | | TITLE ( [  "functional vision" function OR activities OR behaviors OR task]  AND  [child  OR  adolescence  OR adolescent OR pediatric  OR  youth  OR  young] )  AND  ( LIMIT-TO ( DOCTYPE ,  "ar" ) OR  LIMIT- TO ( DOCTYPE ,  "re" ) )  AND  ( LIMIT-TO ( LANGUAGE ,  "English" ) ) AND  ( LIMIT-TO ( SRCTYPE ,  "j" ) ) | 24 |
| Web of Science | | ((TI=("low vision" OR "visual impairment" OR blind OR "visual disability" )) AND TI=(child OR pediatric OR youth OR young* OR adolescence OR adolescent)) AND TI=("functional vision" OR behaviors OR function OR activities OR task) | 195 |
| Cochrane | | ("functional vision" OR behaviors OR function OR activities OR task):ti AND (child OR pediatric OR youth OR young* OR adolescence OR adolescent):ti AND ("low vision" OR "visual impairment" OR blind OR "visual disability"):ti | 37 |
| Google Scholar | | allintitle:["functional vision" OR function OR activities OR behaviors OR task] AND [child OR adolescence OR adolescent OR pediatric OR youth OR young] AND ["low vision" OR "visual impairment" OR blind OR "visual disability"] | 62 |
| Gray literature | <https://greymatters.cadth.ca/> | ["functional vision" OR function OR activities OR behaviors OR task] AND [child OR adolescence OR adolescent OR pediatric OR youth OR young] AND ["low vision" OR "visual impairment" OR blind OR "visual disability"] | 10 |

Study Selection and Data Extraction

Supplementary File 2: Preferred Reporting Items for Systematic reviews and Meta-Analyses extension for Scoping Reviews (PRISMA-ScR) Checklist

| **SECTION** | **ITEM** | **PRISMA-ScR CHECKLIST ITEM** | **REPORTED ON PAGE #** |
| --- | --- | --- | --- |
| **TITLE** | | | |
| Title | 1 | Identify the report as a scoping review. | Page 1 |
| **ABSTRACT** | | | |
| Structured summary | 2 | Provide a structured summary that includes (as applicable): background, objectives, eligibility criteria, sources of evidence, charting methods, results, and conclusions that relate to the review questions and objectives. | Page 1, lines 9-10 |
| **INTRODUCTION** | | | |
| Rationale | 3 | Describe the rationale for the review in the context of what is already known. Explain why the review questions/objectives lend themselves to a scoping review approach. | Page 3 and 4 |
| Objectives | 4 | Provide an explicit statement of the questions and objectives being addressed with reference to their key elements (e.g., population or participants, concepts, and context) or other relevant key elements used to conceptualize the review questions and/or objectives. | Page 4 |
| **METHODS** | | | |
| Protocol and registration | 5 | Indicate whether a review protocol exists; state if and where it can be accessed (e.g., a Web address); and if available, provide registration information, including the registration number. | - |
| Eligibility criteria | 6 | Specify characteristics of the sources of evidence used as eligibility criteria (e.g., years considered, language, and publication status), and provide a rationale. | Page 5, table 1 |
| Information sources* | 7 | Describe all information sources in the search (e.g., databases with dates of coverage and contact with authors to identify additional sources), as well as the date the most recent search was executed. | Page 6, data collection |
| Search | 8 | Present the full electronic search strategy for at least 1 database, including any limits used, such that it could be repeated. | Page 6, Supplementary File 1 |
| Selection of sources of evidence† | 9 | State the process for selecting sources of evidence (i.e., screening and eligibility) included in the scoping review. | Page 6, data collection |
| Data charting process‡ | 10 | Describe the methods of charting data from the included sources of evidence (e.g., calibrated forms or forms that have been tested by the team before their use, and whether data charting was done independently or in duplicate) and any processes for obtaining and confirming data from investigators. | Page 6, data collection |
| Data items | 11 | List and define all variables for which data were sought and any assumptions and simplifications made. | Page 6, data collection |
| Critical appraisal of individual sources of evidence§ | 12 | If done, provide a rationale for conducting a critical appraisal of included sources of evidence; describe the methods used and how this information was used in any data synthesis (if appropriate). | Page 6, data collection |
| Synthesis of results | 13 | Describe the methods of handling and summarizing the data that were charted. | Page 7, data analysis |
| **RESULTS** | | | |
| Selection of sources of evidence | 14 | Give numbers of sources of evidence screened, assessed for eligibility, and included in the review, with reasons for exclusions at each stage, ideally using a flow diagram. | Page 8, figure 2 |
| Characteristics of sources of evidence | 15 | For each source of evidence, present characteristics for which data were charted and provide the citations. | Supplementary Table 5 |
| Critical appraisal within sources of evidence | 16 | If done, present data on critical appraisal of included sources of evidence (see item 12). | We did not conduct critical appraisal |
| Results of individual sources of evidence | 17 | For each included source of evidence, present the relevant data that were charted that relate to the review questions and objectives. | Section 3-2. Vision-related tasks in the children with visual impairment, page 9-17. |
| Synthesis of results | 18 | Summarize and/or present the charting results as they relate to the review questions and objectives. | Section 3-2. Vision-related tasks in the children with visual impairment, page 9-17 |
| **DISCUSSION** | | | |
| Summary of evidence | 19 | Summarize the main results (including an overview of concepts, themes, and types of evidence available), link to the review questions and objectives, and consider the relevance to key groups. | Discussion, page 19-22 |
| Limitations | 20 | Discuss the limitations of the scoping review process. | Limitation, page 22 |
| Conclusions | 21 | Provide a general interpretation of the results with respect to the review questions and objectives, as well as potential implications and/or next steps. | Conclusion, page 23 |
| **FUNDING** | | | |
| Funding | 22 | Describe sources of funding for the included sources of evidence, as well as sources of funding for the scoping review. Describe the role of the funders of the scoping review. | Ethical Considerations, page 23 |

Supplementary File 3: The semi-structured interview form

| 1. May you explain the vision-related tasks that visually impaired children do daily? 2. What types of vison-related tasks are there in activities of daily living, instrumental activities of daily living, education, play, and communication? 3. Are you aware of any other vison-related tasks in children with visual impairment that are not included in the above categories? |
| --- |

Supplementary File 4: The Consolidated Criteria for Qualitative Reporting Research (COREQ) Checklist

| **No. Item** | **Guide questions/description** | **Reported on Page #** |
| --- | --- | --- |
| Domain 1: Research team and reﬂexivity |  |  |
| Personal Characteristics |  |  |
| 1. Inter viewer/facilitator | Which author/s conducted the interview or focus group? | Page 5 / paragraph 2 |
| 2. Credentials | What were the researcher’s credentials? E.g. PhD, MD | Page 5 / paragraph 2 |
| 3. Occupation | What was their occupation at the time of the study? | Page 5 / paragraph2 |
| 4. Gender | Was the researcher male or female? | Page 5 / paragraph2 |
| 5. Experience and training | What experience or training did the researcher have? | Page 5 / paragraph2 |
| Relationship with participants |  |  |
| 6. Relationship established | Was a relationship established prior to study commencement? | Page 5 / paragraph2 |
| 7. Participant knowledge of the interviewer | What did the participants know about the researcher? e.g. personal goals, reasons for doing the research | Page 5 / paragraph2 |
| 8. Interviewer characteristics | What characteristics were reported about the interviewer/facilitator? e.g. Bias, assumptions, reasons and interests in the research topic | Page 5 / paragraph2 |

| Domain 2: study design |  |  |
| --- | --- | --- |
| Theoretical framework |  |  |
| 9. Methodological orientation and Theory | What methodological orientation was stated to underpin the study? e.g. grounded theory, discourse analysis, ethnography, phenomenology, content analysis | Page 3/ last paragraph2 |
| Participant selection |  |  |
| 10. Sampling | How were participants selected? e.g. purposive, convenience, consecutive, snowball | Page 4/ paragraph 1 |
| 11. Method of approach | How were participants approached? e.g. face-to-face, telephone, mail, email | Page 5 / paragraph2 |
| 12. Sample size | How many participants were in the study? | Page 8 / paragraph 1 |
| 13. Non-participation | How many people refused to participate or dropped out? Reasons? | None |
| Setting |  |  |
| 14. Setting of data collection | Where was the data collected? e.g. home, clinic, workplace | Meeting room in a public community center. |
| 15. Presence of non-participants | Was anyone else present besides the participants and researchers? | No |
| 16. Description of sample | What are the important characteristics of the sample? e.g. demographic data, date | Page 8 / paragraph 1 |
| Data collection |  |  |
| 17. Interview guide | Were questions, prompts, guides provided by the authors? Was it pilot tested? | Page 5 / paragraph2 |
| 18. Repeat interviews | Were repeat inter views carried out? If yes, how many? | No |
| 19. Audio/visual recording | Did the research use audio or visual recording to collect the data? | Page 5 / paragraph2 |
| 20. Field notes | Were ﬁeld notes made during and/or after the inter view or focus group? | Page 5 / paragraph2 |
| 21. Duration | What was the duration of the inter views or focus group? | Page 5 / paragraph2 |
| 22. Data saturation | Was data saturation discussed? | Page 5 / paragraph2 |
| 23. Transcripts returned | Were transcripts returned to participants for comment and/or correction? | No |
| Domain 3: analysis and ﬁndings |  |  |
| Data analysis |  |  |
| 24. Number of data coders | How many data coders coded the data? | Page 5 / paragraph 3 |
| 25. Description of the coding tree | Did authors provide a description of the coding tree? | Page 5 / paragraph 3 |
| 26. Derivation of themes | Were themes identiﬁed in advance or derived from the data? | Page 9 / Table 3 |
| 27. Software | What software, if applicable, was used to manage the data? | Page 5 / paragraph 3 |
| 28. Participant checking | Did participants provide feedback on the ﬁndings? | Yes |
| Reporting |  |  |
| 29. Quotations presented | Were participant quotations presented to illustrate the themes/ﬁndings? Was each quotation identiﬁed? e.g. participant number | Page 9 -13 |
| 30. Data and ﬁndings consistent | Was there consistency between the data presented and the ﬁndings? | Yes |
| 31. Clarity of major themes | Were major themes clearly presented in the ﬁndings? | Yes |
| 32. Clarity of minor themes | Is there a description of diverse cases or discussion of minor themes? | Yes |

Supplementary File 5: Characteristics of included studies

| **Author, year** | **Country** | **Type of Study** | **Sample Size** | **Age (year)** | **Visual status** | **Type of instrument** |
| --- | --- | --- | --- | --- | --- | --- |
| Kaiser JT, 2017 (Kaiser & Herzberg, 2017) | United States & Canada | Cross-sectional | 312 | 6-18 | Moderate & severe visual impairment | Functional Vision assessment |
| Elsman EB, 2019(Elsman et al., 2019) | United Kingdom | Psychometric Analysis, Cross-cultural validation in the Dutch | 253 | 7–17 | worse than 20/70 | Functional Vision Questionnaire for Children and Young People |
| Robertson AO, 2020(Robertson et al., 2020) | United kingdom | Questionnaire development | 209 | 8-18 | worse than 20/70 | Functional Vision Questionnaire for Children and Young People |
| Tadić V, 2013 (Tadić et al., 2013) | United kingdom | Questionnaire development | 94 | 10-15 | worse than 20/70 | Functional Vision Questionnaire for Children and Young People |
| Gothwal VK, 2003(Gothwal et al., 2003) | India | Questionnaire development | 78 | 8-18 | Moderate & severe visual impairment | The LV Prasad-Functional Vision Questionnaire |
| Tunay ZÖ, 2016(Tunay et al., 2016) | Turkey | Cross-cultural adaptation & Psychometric analysis | 150 | 6-18 | 20/200-20/1600 | The Cardiff Visual Ability Questionnaire for Children |
| Khadka J, 2010(Khadka et al., 2010) | United kingdom | Questionnaire development | 154 | 5-18 | Moderate & severe visual impairment | Cardiff Visual Ability Questionnaire for Children |
| Çakmak S, 2016(Çakmak et al., 2016) | Turkey | Cross-sectional | 282 | 7-16 | Moderate & severe visual impairment | Gazi Functional Vision Assessment Tool |
| Pitakova I, 2018(Pitakova & Zlatarova, 2018) | Bulgaria | Cross-sectional | 23 | 7-13 | Moderate & severe visual impairment | Adapted The LV Prasad-Functional Vision Questionnaire |
| Gothwal VK, 2012(Gothwal et al., 2012) | India | Questionnaire development | 25 | 8-16 | Moderate & severe visual impairment | The LV Prasad-Functional Vision Questionnaire 2 |
| Castañeda YS, 2016(Castañeda et al., 2016) | United state | Cross-sectional | 16 | 5–17 | Moderate & severe visual impairment | Functional Vision concerns |
| Hatt SR, 2019(Hatt et al., 2019) | United state | Questionnaire development | 480 | 0-17 | Moderate & severe visual impairment | The Pediatric Eye **Questionnaire** |
| Tadić V, 2017(Tadić et al., 2017) | United kingdom | Cross-sectional | 99 | 10-15 | 20/70-20/400 | Functional Vision Questionnaire for Children and Young People |
| Tailor V, 2017(Tailor et al., 2017) | United kingdom | Cross-sectional | 72 | 2–16 | Moderate & severe visual impairment | Cardiff Visual Ability Questionnaire for Children |
| Dahlmann-Noor A, 2018(Dahlmann-Noor et al., 2018) | United kingdom | Cross-sectional | 45 | 2-16 | Moderate & severe visual impairment | Cardiff Visual Ability Questionnaire for Children |
| Dahlmann-Noor A, 2017(Dahlmann-Noor et al., 2017) | United kingdom | Cross-sectional | 119 | 2-16 | Moderate & severe visual impairment | Cardiff Visual Ability Questionnaire for Children |
| Nirmalan PK, 2004(Nirmalan et al., 2004) | India | Cross-sectional | 1194 | 7-15 | lower or equal 20/40 in the worse eye, better than 20/40 in the better eye | The LV Prasad-Functional Vision Questionnaire |
| Hatt SR, 2020(Hatt et al., 2020) | United state | Cross-sectional | 91 | 0-17 | Moderate & severe visual impairment | The Pediatric Eye Questionnaire |
| Leske DA, 2019(Leske et al., 2019) | United state | Questionnaire validation | 48 | 0-17 | Moderate & severe visual impairment | The Pediatric Eye Questionnaire |
| Robertsson A, 2021(Robertson et al., 2021) | United kingdom | Cross-sectional | 152 | 7-18 | Moderate & severe visual impairment | Functional Vision Questionnaire for Children and Young People |
| Leske D, 2021(Leske et al., 2021) | United state | Cross-sectional | 1037 | 0-17 | Moderate & severe visual impairment | The Pediatric Eye Questionnaire |
| Robertsson A, 2022 (Robertson et al., 2022) | United kingdom | Cross-sectional | 93 | 8-18 | Moderate & severe visual impairment | Functional Vision Questionnaire for Children and Young People |
| Smyth, C. A, 214 (Catherine A. Smyth, 2014) | USA | Qualitative study | 30 | from ages 3 months to 3 years | Moderate & severe visual impairment | Interview |
